# Structural Drivers of Cutaneous Leishmaniasis: Examining How the Converging Effects of Displacement, Environmental Disruption, and Political Instability Reshape Epidemiology Beyond Endemic Regions

**DOI:** 10.3390/tropicalmed10090245

**Published:** 2025-08-28

**Authors:** Janice Kim, Tarek Zieneldien, Sophia Ma, Bernard A. Cohen

**Affiliations:** 1 College of Osteopathic Medicine, Michigan State University, East Lansing, MI 48824, USA; 2 School of Medicine, Johns Hopkins University, Baltimore, MD 21205, USA; 3 Department of Dermatology, The Johns Hopkins Hospital, Baltimore, MD 21287, USA

**Keywords:** cutaneous leishmaniasis, global health, climate change, cross-regional epidemiology, refugee health, neglected tropical disease

## Abstract

Cutaneous leishmaniasis (CL) is a vector-borne parasitic disease caused by protozoa of the *Leishmania* genus. Once confined to endemic regions such as the Middle East, Americas, North Africa, and Central Asia, CL is increasingly emerging in non-endemic areas due to a multitude of drivers, including population displacement, environmental disruption, and political instability. These overlapping drivers contribute to expanding sand fly habitats, degrading living conditions, and weakening health systems, increasing transmission. Rising global temperatures further facilitate vector expansion into new regions, where clinical unfamiliarity often leads to misdiagnosis, delayed treatment, increased morbidity, and greater financial burden. Despite its rising incidence and global spread, CL remains a neglected tropical disease since it is seldom fatal, with scant interest by public health authorities and financial donors, limiting activities that further research and prevent spread of the disease. This review synthesizes current evidence on how geopolitical instability, forced migration, and climate-driven ecological changes collectively reshape CL epidemiology and complicate diagnosis, treatment, and surveillance. As CL extends beyond traditional geographic boundaries, it requires integrated strategies that address its multifaceted drivers through strengthened cross-border surveillance, provider education, and international coordination—focusing on prevention, diagnosis, and equitable access to diagnostics and therapeutics, especially among displaced and underserved populations.

## 1. Introduction

**Figure 1:**
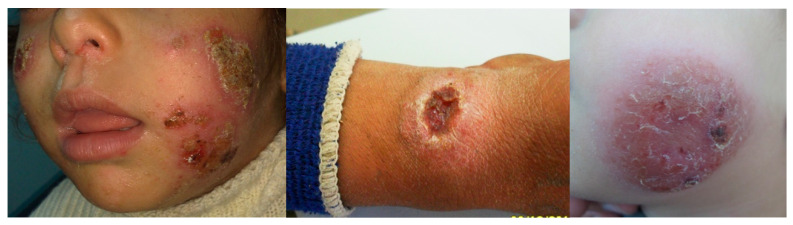
Clinical images of Middle Eastern pediatric patients, illustrating the characteristic presentation of CL with plaques-type and ulcerative lesions.

Leishmaniasis is a vector-borne disease caused by protozoan parasites of the genus *Leishmania*, transmitted to humans infected via female *Phlebotomus* and *Lutzomyia* sand fly bites [1]. The World Health Organization (WHO) has designated leishmaniasis as a neglected tropical disease with significant public health implications. There are many forms of leishmaniasis, the most common form being cutaneous leishmaniasis (CL), which includes hemisphere-specific subtypes such as diffuse and disseminated CL, as well as other forms including mucocutaneous and visceral leishmaniasis [2]. Cutaneous leishmaniasis presents with skin lesions that typically develop within several weeks or months after exposure to bites, evolving from papules to nodular plaques to ulcerative lesions, and can persist for months and sometimes years (Figure 1) [3]. Historically, leishmaniasis has been recognized for centuries. Descriptions of ulcerative skin lesions consistent with CL have appeared in ancient texts from Mesopotamia and Egypt, long before the causative organism was identified such as references dating back to 2000 BCE in the Ebers Papyrus, where CL was likely described as “Nile furuncle” [4]. The disease has also been known by a variety of regional names that reflect both the geographical spread and sociocultural impact, such as “Baghdad boil”, “Balkh sore”, “Jericho button”, and “Aleppo boil” in various regions [4]. These names, while historically and culturally meaningful, have also reflected the wide geographic distribution and deep cultural roots of CL. While this disease is historically endemic to certain regions in the Middle East, North Africa, Central Asia, and parts of Latin America, its epidemiological profile has changed significantly due to the influence of global migration, conflict-driven displacement, and travel [5]. The impact of these changes has challenged conventional geographic boundaries of disease transmission resulting in the increased incidence of CL in previously non-endemic areas, particularly in fragile health systems and refugee-hosting nations.

To explore this evolving landscape, relevant articles were identified through systematic searches of PubMed, Google Scholar, and other academic databases (2000–2025) using search terms such as “cutaneous leishmaniasis”, or “*Leishmania*”, and “epidemiology”, “clinical management”, “treatment resistance”, “diagnosis”, “migration”, “climate change”, “vector control”, “public health policy”, and “surveillance systems.” Articles were included if they addressed key themes such as epidemiologic trends and geographic distribution of CL, clinical presentation, and therapeutic approaches, including diagnostic challenges and treatment resistance, the impact of migration and displacement on disease dynamics, environmental and ecological determinants of transmission, public health surveillance and control strategies, and policy and health system responses to emerging challenges. Articles not primarily focused on CL, non-English publications, and studies lacking relevance to global or regional public health implications were excluded. Over 88 articles were selected, including epidemiological research, systematic reviews, clinical trials, public health reports, and policy analyses.

By examining migration-driven outbreaks, travel-related risks, and climate impacts across regions, this review synthesizes recent evidence on the evolving epidemiology of CL amid displacement, geopolitical instability, environmental disruption, and climate change. While previous clinical and regional reviews have provided valuable insights into the diagnosis, treatment, and management of CL within refugee and traveler populations, this article offers a complementary structural perspective. It explores the intersecting drivers reshaping CL beyond traditional endemic areas, including novel transmission patterns, climate-driven shifts in vectors and reservoirs, and unique challenges faced in non-endemic and displaced settings (Table 1). Furthermore, it addresses emerging gaps in public health and surveillance resulting from the geographic expansion of CL. By integrating clinical, ecological, and policy perspectives, the review aims to guide research and multisectoral strategies that strengthen surveillance, provider education, and equitable access to care for affected populations. 

## 2. A Global Perspective: Cross-Regional Comparisons

**Figure 2:**
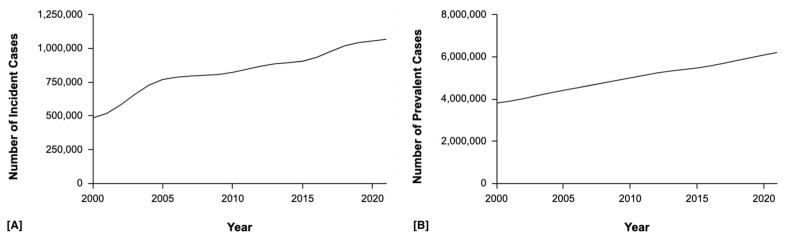
Global trends in (**A**) incident cases and (**B**) prevalent cases of cutaneous and mucocutaneous leishmaniasis from 2000 to 2021. Data represent absolute case counts obtained from the publicly accessible 2021 Global Burden of Disease (GBD) study [17].

CL presents with significant clinical, ecological, and therapeutic diversity across regions, highlighting the importance of cross-regional comparisons. Old World CL is prevalent in countries such as Syria, Iran, and Afghanistan and the disease is most commonly caused by *L. tropica* and *L. major* [6]. Infection from these species causes dry or wet ulcerative lesions and transmission can be anthroponotic or zoonotic depending on the setting [7]. In Africa, *L. major* predominates across North and East Africa, while *L. aethiopica* is responsible for more severe and diffuse forms of the CL [8]. In Europe, particularly in southern and increasingly more central regions such as Italy and Spain, *L. infantum* has emerged as a cause of CL [9]. In the Americas, CL is primarily caused by *L. braziliensis*, *L. panamensis*, and *L. guyanensis* in South and Central America whereas in Mexico CL is caused by *L. mexicana* and *L. amazonensis* [10]. Treatment of CL is complex and depends on the species involved and area of the infection, and treatment may involve topical or systemic therapy [11,12]. Oral azole antifungal drugs, such as fluconazole and itraconazole, have been used as treatment options for CL [13]. Their efficacy varies by *Leishmania* species and geographic region, with fluconazole demonstrating benefit against *L. major* in the Old World [14]. However, long-term treatment with azoles have toxicity risks including liver toxicity and QTc prolongations [14]. The most commonly prescribed treatment for Old World CL is antimonials [15]. In contrast, New World CL is seen in Brazil and other parts of Latin America and the most prevalent species include *L. braziliensis* and *L. guyanensis* which are known to lead to mucocutaneous disease with the potential to manifest systemically [16]. Over the past two decades, both the prevalence and incidence of both cutaneous and mucocutaneous leishmaniasis have risen worldwide (Figure 2) [17]. Treatment for New World CL includes liposomal amphotericin B or miltefosine [18]. 

Migration and international travel represent two pathways through which CL can spread across borders. Forced migration, especially in regions experiencing armed conflict or political instability can lead to sustained transmission of CL as displayed populations carry the infection into new geographic areas [19]. This has been particularly evident in the Middle East and Central Asia where refugees and internally displaced people have contributed to the re-emergence of CL in previously controlled or non-endemic zones [19]. In contrast, short-term international travel creates a different challenge in the spread of CL. Travelers to endemic areas such as South America, Africa, or Asia may acquire the disease during brief exposure and return to non-endemic countries where diagnostic suspicion is low, often resulting in delayed treatment and increased risk of complications [20]. Migrants may serve as reservoirs for transmission while travelers present isolated imported cases of CL, both highlighting the need for increased surveillance and education strategies to address these distinct pathways of CL infection. 

## 3. Migration-Driven Dynamics: Geopolitical Instability and Displacement in Syria and Neighboring Regions

Syria has experienced over a decade of armed conflict which has devastated the healthcare system and displaced over 12 million people both internally and across borders [21]. This mass displacement of individuals has facilitated the spread of *Leishmania tropica* (*L. tropica*) and *Leishmania major* (*L. major*) into neighboring countries such as Jordan, a country hosting one of the highest refugee population per capita, Lebanon, and Turkey [22]. *L. tropica* accounts for up to 85% of reported CL cases in Syrian refugee camps in Lebanon and Jordan, where vector exposure is heightened by overcrowded living conditions, open sewage, and poor housing structures that hinder effective vector control [23]. Additionally, the disruption of vector surveillance and public health systems in conflict zones has created a “leishmaniasis corridor” spanning from Aleppo, Syria through northern Syria into refugee-hosting border regions [24]. This surge is exacerbated by the widespread presence of sandflies and the movement of displaced populations, who serve as human reservoirs and increase the risk of introducing this disease to previously non-endemic areas [25].

## 4. Cross-Border Epidemiological Trends

Countries such as Lebanon and Turkey, which previously reported low or sporadic incidences of CL have experienced surges in CL cases since 2013, following the influx of Syrian refugees as a result of the Syrian conflict [26]. In Lebanon, the number of reported cases increased from fewer than a dozen per year to over a thousand within a year of the Syrian crisis, particularly in informal settlements in the Bekaa Valley [27]. Likewise, in Jordan’s Zaatari refugee camp, sustained CL transmission has been reported, driven by ongoing human-vector contact and inadequate vector control measures, with 92.1% of cases classified as imported infections [28]. In Turkey, provinces such as Gaziantep and Sanliurfa in which CL was previously uncommon have similarly experienced significant increases in CL following the arrival of Syrian refugees [29]. As populations move between areas that are endemic and non-endemic areas, they can re-introduce CL into areas that had previously controlled the disease or eradicated it [30]. This migration epidemiological shift has made CL not only a localized concern, but also a transboundary public health issue that requires coordination across regions [31]. 

Additionally, the urban spread of *L. tropica* and the rural spread of *L. major* reflects distinct ecological dynamics. Both Afghanistan and its neighboring countries in Central Asia and the Middle East are hotspots for CL with *L. tropica* predominating in urban areas and *L. major* in rural areas [23,32]. Migration across borders, driven by regional instability or economic displacement has contributed to the sustained transmission and geographic spread of both types of CL [30]. In urban centers such as Kabul, *L. tropica* is the dominant species and is able to spread efficiently due to the densely populated environment where sandfly vectors are able to thrive because of poor sanitation and informal housing [33]. On the other hand, *L. major* is dominated in rural agricultural zones, where transmission cycles involving rodents are more prevalent, compared to sandflies [34,35]. The primary vectors species involved include *Phlembotomus papatasi* for *L. major* and *Phlebotommus sergenti* for *L. tropica*, each exhibiting distinct ecological preferences. Rodents serve as major animal reservoirs for *L. major* maintaining the zoonotic cycle, while *L. tropica*, humans serve as the primary reservoir [36].

Beyond the Middle East and Central Asia, CL is increasingly reported in Latin America and the southern United States, where it has been spreading northwards, with endemic transmission observed in countries such as Colombia, Peru, and Brazil due to the climatic, geographic, and social conditions of these regions—particularly among rural populations, children, and Indigenous communities [37,38,39,40]. In northern Texas, multiple pediatric cases of locally acquired *L. mexicana* have been reported in patients with no travel history outside the region and without infected family members, suggesting that CL is now emerging in areas of the U.S. previously considered non-endemic [39]. Furthermore, two horses in Florida with no history of travel were diagnosed with CL caused by a *Leishmania* species from the *Mundinia* subgenus, which has been linked to human infections in other countries, highlighting the potential for zoonotic transmission and further geographic expansion of leishmaniasis in the United States [41,42].

As populations migrate between rural and urban environments, often due to conflict, economic instability, or internal displacement, these distinct transmission patterns begin to blur [23]. Shifts in land use, urbanization, and increased environmental degradation have resulted in increased adaptation and spread of vector populations into new environments. For example, sand flies have been increasingly detected in peri-urban and urban settings, including construction zones and refugee camps, where control efforts are difficult to sustain. This rural-to-urban shift in transmission highlights how human movement can contribute to the reshaping of epidemiological landscapes, complicating vector control efforts. Current challenges in vector control include lack of targeted interventions for sand fly populations, lack of funding, insecticide resistance, fragile health infrastructure in conflict zones of endemic areas, and decreased public health priorities [25,43]. The marked increase in CL across previously non-endemic regions highlights how forced migration and conflict-driven displacement are transforming CL from a localized endemic disease into a cross-border public health concern with growing implications on global surveillance.

## 5. Clinical Relevance in Refugee Settings

The clinical management of CL in refugee populations presents many unique challenges, especially when caused by *L. Tropica*. Unlike *L. major*, which is resolved more readily, *L. tropica* creates chronic, relapsing lesions that do not respond well to first line treatments [44]. CL typically manifests as painless papules or nodules at the site of the sand fly bite, which may ulcerate over time and evolve into chronic plaques or crusted lesions [45]. Additionally, since CL is non-endemic to these refugee-hosting countries, there is limited availability of antileishmanial drugs such as pentavalent antimonials and liposomal amphotericin, hindering effective management [46].

Similarly, many patients face significant delays in diagnosis due to poor diagnostic capacity, lack of familiarity with the disease among healthcare professionals, and symptom overlap with other dermatologic or infectious diseases [47]. In non-endemic regions, particularly in refugee hosting countries where CL is unfamiliar, diagnosis is frequently delayed due to several systemic barriers [48]. Healthcare professionals may have limited clinical exposure to CL, and its presentation can mimic other dermatologic or infectious diseases such as bacterial ulcers, fungal infections, or skin cancers [49]. Diagnostic capacity is also often limited to clinical suspicion or non-specific histopathology as few centers are equipped with diagnostic tools like PCR or culture of *Leishmania* organisms to confirm diagnosis [50]. These barriers are compounded by language and cultural barriers between patients and providers, further delaying care.

Moreover, treatment for CL is long, requiring careful monitoring that is not feasible in resource-limited settings such as refugee camps [10]. Refugee health systems are often overburdened and underfunded, leaving them ill-equipped to address CL, a multifaceted disease that requires both clinically and psychological care, leading to high morbidity and poor long-term outcomes of affected individuals [51,52]. These therapeutic regimens for CL often span weeks, requiring follow-up visits and monitoring that are difficult to maintain in refugee camps.

## 6. Travel-Acquired Cutaneous Leishmaniasis: Brazil as a Hotspot

In the Americas, Brazil consistently reports the highest number of CL cases, averaging approximately 34,954 cases annually [53]. This high disease burden is due to the country’s vast ecological diversity and the presence of multiple *Leishmania* species particularly *L. braziliensis* and *L. guyanensis* [54]. These species contribute to the complexity of zoonotic transmission patterns, resulting in increased difficulty of both surveillance and clinical management [55]. Tourists visiting endemic regions, especially the Amazon rainforest and surrounding forested areas, face risks of New World CL variants [56]. These variants have different clinical presentations and can potentially progress to mucocutaneous leishmaniasis, which requires prolonged and specialized treatment [56]. The regional diversity of CL and the transmission dynamics reinforces the importance of public health messaging and awareness for travelers visiting these high-risk areas. Preventive care, such as the use of appropriate clothing that minimizes skin exposure, the use of repellents such as Picaridin or DEET, and the use of bed nets can be used by visitors to lessen contact with mosquitoes that may serve as vectors in large ecotourist attractions such as The Reserva Particular do Patrimônio Natural Santuário do Caraça [57]. The sanctuary, which comprises 11,233 hectares of environmental preservation in the State of Minas Gerais, has also successfully utilized traps for small mammals, preventing their circulation in lodging areas [57]. Further studies in similar areas are crucial for monitoring infections in global tourist attractions, which potentially serve as migration routes for the disease in non-endemic areas. 

Control of CL is further complicated by intricate zoonotic transmission involving a wide range of region-specific animal reservoirs and sand fly vectors. Mammalian hosts such as rodents and sloths serve as natural reservoirs, maintaining the parasite environments and making eradication difficult [58]. Additionally, sand fly vectors are highly adapted to both forested areas and peri-urban habitats, facilitating the transmission to animals and humans [59]. Moreover, as eco-tourism expands into previously undisturbed environments, there is an increased risk for travelers to encounter these reservoirs and vectors [60]. The mobility of tourists combined with limited awareness of transmission risks presents a challenge for both preventative measures and post-travel diagnosis in non-endemic countries [61].

## 7. Climate Change as a Catalyst

Emerging evidence suggests that climate change has also played a growing role in the expansion of sand fly habitats globally. The rising temperatures, changes in precipitation patterns, and land degradation have expanded the range of suitable habitats for sandflies, potentially increasing CL transmission risks in non-endemic regions [62,63]. As forests are cleared and temperatures rise, it creates a suitable environment for sand fly survival and reproduction which expands into new ecological zones, increasing the overall transmission potential of CL [64]. These shifts intensify the disease in traditional endemic areas but also increase the risk of CL emerging in previously unaffected areas [65]. For instance, milder winters and longer summers can extend sand fly breeding seasons, while increased humidity can enhance larval development, leading to higher vector densities [66]. Additionally, recent multi-site analyses have demonstrated that moderate increases in rainfall can promote higher sand fly densities by increasing the availability of moist, organic-rich resting and breeding sites that support larval development, thereby enhancing sand fly survival and reproduction, further supporting the role of shifting precipitation patterns in vector expansion [66].

Areas that were previously too cold or arid for sandfly survival are becoming more suitable, raising the possibility of CL outbreaks in parts of southern Europe, North America, and Central Asia [67,68]. Leishmaniasis is spreading to new areas, including parts of southern and central Europe. For example, in a recent study conducted in Emilia-Romagna, a traditionally endemic region in Northern Italy, identified active circulation of *Leishmania infantum* in local *Phlebotomus* sand fly populations [69]. The study also reported the emergence of insecticide resistance mutations, raising concerns about the sustainability of current vector control efforts, these findings suggest not only a persistent presence, but a potential expansion of leishmaniasis risk zones in Italy [69]. Similar trends have been observed in Spain, Greece, and the Balkans, where warmer temperatures and changes in vector-host interactions have been linked to sporadic CL and visceral leishmaniasis cases [62,70]. Climate predictors, such as wind speed also have an effect, with wind speeds < 10 km/h enabling sand fly flight without dispersing them beyond endemic zones, while speeds > 10 km/h reduce biting opportunities and decrease cases [68].

Additionally, human-driven factors, including urbanization, agricultural expansion, and deforestation are accelerating ecological disturbances and result in closer contact with sand fly vectors and animal reservoirs [65]. For example, sandflies have been increasingly detected in peri-urban construction sites, waste accumulation zones, and areas with poor drainage, highlighting the intersection of environmental change and vector adaptation of CL [71,72]. To illustrate, in Iran, human modifications such as asbestos cement roofs have been found to increase household transmission odds 4.77 × when compared to individuals living in bituminous roofs [73]. Furthermore, natural catastrophes and extreme weather events, such as droughts, floods, or earthquakes also can increase the risk of CL, as residents are often forced to leave their homes and reside in crowded conditions, while the ecological disturbances create favorable conditions for sand fly breeding and increase human-vector contact [74]. Following the 2003 earthquake in southeastern Iran, a new outbreak of cutaneous leishmaniasis caused by *Leishmania tropica* was reported [75]. Longitudinal studies have since supported the sustained rise in leishmaniasis transmission risk to the earthquake’s long term-effects [76]. Since West Asia is among the most tectonically active regions globally, the occurrence of similarly devastating earthquakes remains a strong possibility [77]. These environmental changes result in unstable transmission cycles and make predicting and preventing future outbreaks more complex. This is particularly concerning in areas where surveillance systems are weak or nonexistent, and where health systems are ill-equipped to detect early cases or deploy timely interventions [78]. Consequently, early outbreak detection, proactive vector control strategies, and enhanced surveillance of rural and peri-urban communities vulnerable to ecological disruption is needed [60]. These trends mirror broader patterns where climate change, urbanization, and increased human–animal interaction have facilitated the spread of sand fly vectors into previously unaffected regions.

## 8. Public Health Implications

The evolving epidemiology of CL presents challenges for public health systems globally, particularly in under-resourced endemic regions and host countries for displaced populations or returning travelers [79]. Surveillance systems in many endemic countries remain fragmented or outdated, making it difficult to detect emerging trends or conduct timely outbreak response [76]. For example, in sub-Saharan Africa, CL is not a notifiable disease in many countries, making it a significant gap on the epidemiological burden map of CL [80]. In contrast, a surveillance system covering the South of France, French West Indies, and French Guiana, implemented since 1998, has enabled better epidemiological monitoring of both autochthonous and imported CL cases [81]. This demonstrates the value of such systems through shedding light on transmission dynamics, thereby helping inform control strategies. Additionally, non-endemic countries lack clinical familiarity, diagnostic tools, or medications necessary to manage CL cases, highlighting the importance of collaboration between global health organizations to address the diagnostic, therapeutic, and preventative challenges [82]. The Pan American Health Organization has led efforts to standardize and centralize surveillance for New World CL in Latin America, leading to the development of SisLeish, a regional information system that serves as a critical tool for prioritizing endemic areas [83,84]. Consequently, preventative approaches such as community education, vector control, and climate-related drivers of CL are essential to mitigate the rising cases of CL and prevent its further expansion into new regions. 

Furthermore, addressing the expanding distribution and complex drivers of CL requires integrated, multisectoral strategies beyond traditional methods. Strengthening cross-border data sharing and interoperable surveillance with standardized reporting tailored to migration and climate risks is essential [85]. Incorporating environmental and climate monitoring, such as vector habitat modeling and weather forecasting, can help anticipate transmission shifts [86]. Likewise, fostering multisectoral collaboration among healthcare, migration, environmental, and policy sectors is vital to tackling social and ecological factors fueling CL spread as a whole [87,88]. Further expanding healthcare provider training to include migration dynamics and ecological risks may also better prepare clinicians in new endemic areas. Additionally, sustainable policy frameworks and funding must ensure equitable access to diagnostics, treatment, and surveillance—particularly in refugee-hosting and underserved communities. While traditional vector control, clinical capacity building, and community education remain foundational, embedding these within a comprehensive, anticipatory framework is critical to reduce the spread and impact of CL amid evolving global challenges.

## 9. Conclusions

As migration, international travel, and climate change shifts continue to reshape CL distribution, its rising prevalence in formerly unaffected or minimally impacted areas presents a dynamic and multidimensional global health concern. Addressing this emerging threat requires reconstruction of surveillance systems to enable timely detection, standardized reporting, and cross-border data sharing to monitor and manage new cases in both endemic and non-endemic regions. Clinical management of CL is further complicated by rising treatment resistance, diagnostic challenges, and a lack of familiarity among healthcare providers in newly stricken regions, underscoring the urgent need for targeted education and capacity building, especially in resource-limited migrant settings. Future research should prioritize the characterization of CL epidemiological shifts linked to climate change and zoonotic vectors to improve outbreak prediction and mitigate disease impact. Equally important is fostering multisectoral partnerships that bridge health, environmental, migration, and policy domains are essential to dismantle systemic barriers and address social determinants fueling disease spread. Strengthening global collaboration and policy innovation are essential to reducing the expanding burden of CL among at-risk populations worldwide in an increasingly interconnected and climate-affected world.

## Figures and Tables

**Table 1:** Key drivers of cutaneous leishmaniasis global spread and emergence in non-endemic areas.

Driver Category	Description	Mechanism and Impact	Example
Population Displacement	Forced migration, refugee movements, internal displacement due to conflict and political instability	Overcrowded living conditions, poor sanitation, and open sewage in camps increase exposure to sandflies; displaced populations carry infections to new areas	Syrian refugee camps in Lebanon, Jordan, and Turkey spreading *L. tropica* and *L. major*; “leishmaniasis corridor” from Aleppo
Political Instability & Conflict	Armed conflict and government breakdown disrupt public health and vector control systems	Collapse of healthcare infrastructure, lack of vector surveillance, increased exposure due to damaged housing and poor vector control	Syrian civil war effects resulting in rising CL cases in neighboring countries, weakened surveillance and public health
Environmental & Climate Change	Rising temperatures, altered precipitation, deforestation, urbanization, and natural disasters impacting sand fly habitats	Expansion of sand fly habitats into previously non-endemic zones; prolonged breeding seasons and higher vector densities; insecticide resistance emergence	CL emergence in southern Europe (Italy, Spain), southern US (Texas, Florida), peri-urban sand fly adaptation
Vector and Reservoir Ecology	Different sand fly species (e.g., *Phlebotomus papatasi*, *P. sergenti*) and animal reservoirs, including rodents and horses	Species-specific transmission cycles: urban *L. tropica* (human reservoir) vs. rural *L. major* (rodent reservoir); zoonotic cycles complicate control	Urban spread in Kabul versus rural transmission; US horse infections by *Leishmania* Mundinia species indicating zoonosis
Travel and Globalization	International tourism and short-term travel to endemic regions	Travelers acquire infections and may import cases to non-endemic countries, where clinical suspicion is low, causing delayed diagnosis and treatment	Tourists acquiring CL in the Brazilian Amazon and other Latin America regions; eco-tourism increasing exposure
Health System Limitations	Limited diagnostic capacity, provider unfamiliarity, fragmented surveillance, and resource-constrained health systems in refugee-hosting and non-endemic countries	Delayed or misdiagnosis due to overlapping symptoms and lack of PCR/culture diagnostics; overburdened refugee health services hinder timely treatment and follow-up	Delays in Lebanon, Jordan refugee camps; scarcity of antileishmanial drugs; non-notifiable status in sub-Saharan Africa

## Data Availability

Not applicable.

## Abbreviations

The following abbreviations are used in this manuscript:

CL	Cutaneous leishmaniasis
*L. braziliensis*	*Leishmania braziliensis*
*L. guyanensis*	*Leishmania guyanensis*
*L. major*	*Leishmania major*
*L. tropica*	*Leishmania tropica*
WHO	World Health Organization

## References

[1] World Health Organization (2023). Leishmaniasis WHO Facts Sheets: WHO. https://www.who.int/news-room/fact-sheets/detail/leishmaniasis.

[2] McGwire B.S., Satoskar A.R. (2014). Leishmaniasis: Clinical syndromes and treatment. QJM.

[3] Berman J.D. (1997). Human leishmaniasis: Clinical, diagnostic, and chemotherapeutic developments in the last 10 years. Clin. Infect. Dis..

[4] Nazzaro G., Rovaris M., Veraldi S. (2014). Leishmaniasis: A disease with many names. JAMA Dermatol..

[5] Burza S., Croft S.L., Boelaert M. (2018). Leishmaniasis. Lancet.

[6] Masmoudi A., Hariz W., Marrekchi S., Amouri M., Turki H. (2013). Old World cutaneous leishmaniasis: Diagnosis and treatment. J. Dermatol. Case Rep..

[7] Yadav P., Azam M., Ramesh V., Singh R. (2023). Unusual Observations in Leishmaniasis-An Overview. Pathogens.

[8] Blaizot R., Pasquier G., Kone A.K., Duvignaud A., Demar M. (2024). Cutaneous leishmaniasis in sub-Saharan Africa: A systematic review of Leishmania species, vectors and reservoirs. Parasites Vectors.

[9] Ready P.D. (2010). Leishmaniasis emergence in Europe. Euro Surveill..

[10] de Vries H.J.C., Schallig H.D. (2022). Cutaneous Leishmaniasis: A 2022 Updated Narrative Review into Diagnosis and Management Developments. Am. J. Clin. Dermatol..

[11] González U., Pinart M., Rengifo-Pardo M., Macaya A., Alvar J., Tweed J.A. (2009). Interventions for American cutaneous and mucocutaneous leishmaniasis. Cochrane Database Syst. Rev..

[12] González U., Pinart M., Reveiz L., Alvar J. (2008). Interventions for Old World cutaneous leishmaniasis. Cochrane Database Syst. Rev..

[13] Madusanka R.K., Silva H., Karunaweera N.D. (2022). Treatment of Cutaneous Leishmaniasis and Insights into Species-Specific Responses: A Narrative Review. Infect. Dis. Ther..

[14] Garza-Tovar T.F., Sacriste-Hernández M.I., Juárez-Durán E.R., Arenas R. (2020). An overview of the treatment of cutaneous leishmaniasis. Fac. Rev..

[15] Heras-Mosteiro J., Monge-Maillo B., Pinart M., Lopez Pereira P., Reveiz L., Garcia-Carrasco E., Campuzano Cuadrado P., Royuela A., Mendez Roman I., López-Vélez R. (2017). Interventions for Old World cutaneous leishmaniasis. Cochrane Database Syst. Rev..

[16] Kevric I., Cappel M.A., Keeling J.H. (2015). New World and Old World Leishmania Infections: A Practical Review. Dermatol. Clin..

[17] Institute for Health Metrics and Evaluation (IHME) (2022). Global Burden of Disease Study 2021.

[18] Mitropoulos P., Konidas P., Durkin-Konidas M. (2010). New World cutaneous leishmaniasis: Updated review of current and future diagnosis and treatment. J. Am. Acad. Dermatol..

[19] Bizri N.A., Alam W., Khoury M., Musharrafieh U., Ghosn N., Berri A., Bizri A.R. (2021). The Association Between the Syrian Crisis and Cutaneous Leishmaniasis in Lebanon. Acta Parasitol..

[20] Curtin J.M., Aronson N.E. (2021). Leishmaniasis in the United States: Emerging Issues in a Region of Low Endemicity. Microorganisms.

[21] Haar R., Rayes D., Tappis H., Rubenstein L., Rihawi A., Hamze M., Almhawish N., Wais R., Alahmad H., Burbach R. (2024). The cascading impacts of attacks on health in Syria: A qualitative study of health system and community impacts. PLoS Glob. Public Health.

[22] Saroufim M., Charafeddine K., Issa G., Khalifeh H., Habib R.H., Berry A., Ghosn N., Rady A., Khalifeh I. (2014). Ongoing epidemic of cutaneous leishmaniasis among Syrian refugees, Lebanon. Emerg. Infect. Dis..

[23] Ghatee M.A., Taylor W.R., Karamian M. (2020). The Geographical Distribution of Cutaneous Leishmaniasis Causative Agents in Iran and Its Neighboring Countries, A Review. Front. Public Health.

[24] Rehman K., Walochnik J., Mischlinger J., Alassil B., Allan R., Ramharter M. (2018). Leishmaniasis in Northern Syria during Civil War. Emerg. Infect. Dis..

[25] Al-Salem W.S., Pigott D.M., Subramaniam K., Haines L.R., Kelly-Hope L., Molyneux D.H., Hay S.I., Acosta-Serrano A. (2016). Cutaneous Leishmaniasis and Conflict in Syria. Emerg. Infect. Dis..

[26] Salam N., Al-Shaqha W.M., Azzi A. (2014). Leishmaniasis in the middle East: Incidence and epidemiology. PLoS Neglected Trop. Dis..

[27] World Health Organization (2022). Lebanon EPI Monitor Eastern Mediterranean Region World Health Organization. https://www.emro.who.int/lbn/information-resources/epi-monitor.html.

[28] Amr Z.S., Kanani K., Shadfan B., Hani R.B. (2018). Cutaneous Leishmaniasis among Syrian Refugees in Jordan: A Retrospective Study. Bull. Soc. Pathol. Exot..

[29] Yentur Doni N., Gurses G., Dikme R., Aksoy M., Yildiz Zeyrek F., Simsek Z., Satoskar A.R., Varikuty S., Yesilova Y. (2020). Cutaneous Leishmaniasis due to Three Leishmania Species Among Syrian Refugees in Sanliurfa, Southeastern Turkey. Acta Parasitol..

[30] Bailey F., Mondragon-Shem K., Hotez P., Ruiz-Postigo J.A., Al-Salem W., Acosta-Serrano Á., Molyneux D.H. (2017). A new perspective on cutaneous leishmaniasis-Implications for global prevalence and burden of disease estimates. PLoS Neglected Trop. Dis..

[31] Karimi T., Sharifi I., Aflatoonian M.R., Aflatoonian B., Mohammadi M.A., Salarkia E., Babaei Z., Zarinkar F., Sharifi F., Hatami N. (2021). A long-lasting emerging epidemic of anthroponotic cutaneous leishmaniasis in southeastern Iran: Population movement and peri-urban settlements as a major risk factor. Parasites Vectors.

[32] Mosawi S.H., Dalimi A. (2016). Molecular detection of Leishmania spp. isolated from cutaneous lesions of patients referred to Herat regional hospital, Afghanistan. East. Mediterr. Health J..

[33] Mirahmadi H., Salimi Khorashad A., Sohrabnahad A., Heydarian P., Bizhani N. (2016). Species Identification and Molecular Typing of Leishmania Spp. Using Targeting HSP70 Gene in Suspected Patients of Cutaneous Leishmaniasis from Sistan and Baluchestan Province, Southeast Iran. Iran. J. Parasitol..

[34] Akhavan A.A., Yaghoobi-Ershadi M.R., Khamesipour A., Mirhendi H., Alimohammadian M.H., Rassi Y., Arandian M.H., Jafari R., Abdoli H., Shareghi N. (2010). Dynamics of Leishmania infection rates in Rhombomys opimus (Rodentia: Gerbillinae) population of an endemic focus of zoonotic cutaneous leishmaniasis in Iran. Bull. Soc. Pathol. Exot..

[35] Karamian M., Ghatee M.A., Shayesteh M., Taylor W.R., Mohebi-Nejad S., Taheri G., Jamavar M.R. (2021). The effect of geo-climatic determinants on the distribution of cutaneous leishmaniasis in a recently emerging focus in eastern Iran. Parasites Vectors.

[36] Mozafari O., Sofizadeh A., Shoraka H.R. (2020). Distribution of Leishmania Infection in Humans, Animal Reservoir Hosts and Sandflies in Golestan Province, Northeastern Iran: A Systematic Review and Meta-Analysis. Iran. J. Public Health.

[37] Medina-Morales D.A., Machado-Duque M.E., Machado-Alba J.E. (2017). Epidemiology of Cutaneous Leishmaniasis in a Colombian Municipality. Am. J. Trop. Med. Hyg..

[38] Hashiguchi Y., Gomez E.A.L., Cáceres A.G., Velez L.N., Villegas N.V., Hashiguchi K., Mimori T., Uezato H., Kato H. (2018). Andean cutaneous leishmaniasis (Andean-CL, uta) in Peru and Ecuador: The causative Leishmania parasites and clinico-epidemiological features. Acta Trop..

[39] Nepal B., McCormick-Baw C., Patel K., Firmani S., Wetzel D.M. (2024). Cutaneous Leishmania mexicana infections in the United States: Defining strains through endemic human pediatric cases in northern Texas. mSphere.

[40] Reis E.S.D., Paz W.S., Santos Ramos R.E., Nunes Ribeiro C.J., Biano L.S., Bezerra-Santos M., de Oliveira C.I., Lipscomb M.W., de Moura T.R. (2024). Spatial and temporal modeling of the global burden of Cutaneous Leishmaniasis in Brazil: A 21-year ecological study. PLoS Neglected Trop. Dis..

[41] Menezes R.C., Campos M.P., Popielarczyk M., Kiupel M. (2019). Cutaneous Leishmaniosis caused by Leishmania martiniquensis in a Horse in Florida. J. Comp. Pathol..

[42] Reuss S.M., Dunbar M.D., Calderwood Mays M.B., Owen J.L., Mallicote M.F., Archer L.L., Wellehan J.F. (2012). Autochthonous Leishmania siamensis in horse, Florida, USA. Emerg. Infect. Dis..

[43] Wilson A.L., Courtenay O., Kelly-Hope L.A., Scott T.W., Takken W., Torr S.J., Lindsay S.W. (2020). The importance of vector control for the control and elimination of vector-borne diseases. PLoS Neglected Trop. Dis..

[44] Kassi M., Kassi M., Afghan A.K., Rehman R., Kasi P.M. (2008). Marring leishmaniasis: The stigmatization and the impact of cutaneous leishmaniasis in Pakistan and Afghanistan. PLoS Neglected Trop. Dis..

[45] Moore E.M., Lockwood D.N. (2011). Leishmaniasis. Clin. Med..

[46] Lindner A.K., Richter J., Gertler M., Nikolaus M., Equihua Martinez G., Müller K., Harms G. (2020). Cutaneous leishmaniasis in refugees from Syria: Complex cases in Berlin 2015–2020. J. Travel Med..

[47] Poloni A., Giacomelli A., Corbellino M., Grande R., Nebuloni M., Rizzardini G., Ridolfo A.L., Antinori S. (2023). Delayed diagnosis among patients with cutaneous and mucocutaneous leishmaniasis. Travel Med. Infect. Dis..

[48] Ekemen S., Nalcaci M., Toz S., Sanjoba C., Demirkesen C., Cetin E.D., Tecimer T., Yildiz P., Gursel M., Ince U. (2024). Diagnostic challenges in cutaneous leishmaniasis due to atypical Leishmania infantum: Pathologists’ insights from re-emergence zones. Front. Med..

[49] Gurel M.S., Tekin B., Uzun S. (2020). Cutaneous leishmaniasis: A great imitator. Clin. Dermatol..

[50] Vega-López F. (2003). Diagnosis of cutaneous leishmaniasis. Curr. Opin. Infect. Dis..

[51] Douba M.D., Abbas O., Wali A., Nassany J., Aouf A., Tibbi M.S., Kibbi A.G., Kurban M. (2012). Chronic cutaneous leishmaniasis, a great mimicker with various clinical presentations: 12 years experience from Aleppo. J. Eur. Acad. Dermatol. Venereol..

[52] Hameed S., Sadiq A., Din A.U. (2018). The Increased Vulnerability of Refugee Population to Mental Health Disorders. Kans. J. Med..

[53] Portella T.P., Kraenkel R.A. (2021). Spatial-temporal pattern of cutaneous leishmaniasis in Brazil. Infect. Dis. Poverty.

[54] Hong A., Zampieri R.A., Shaw J.J., Floeter-Winter L.M., Laranjeira-Silva M.F. (2020). One Health Approach to Leishmaniases: Understanding the Disease Dynamics through Diagnostic Tools. Pathogens.

[55] Heeren S., Sanders M., Shaw J.J., Pinto Brandão-Filho S., Côrtes Boité M., Motta Cantanhêde L., Chourabi K., Maes I., Llanos-Cuentas A., Arevalo J. (2024). Evolutionary genomics of Leishmania braziliensis across the neotropical realm. Commun. Biol..

[56] Mansueto P., Seidita A., Vitale G., Cascio A. (2014). Leishmaniasis in travelers: A literature review. Travel Med. Infect. Dis..

[57] Tonelli G.B., Tanure A., Rego F.D., Carvalho G.M.L., Stumpp R., Ássimos G.R., Campos A.M., Lima A., Gontijo C.M.F., Paz G.F. (2017). Leishmania (Viannia) braziliensis infection in wild small mammals in ecotourism area of Brazil. PLoS ONE.

[58] Claborn D.M. (2010). The biology and control of leishmaniasis vectors. J. Glob. Infect. Dis..

[59] Chen Q., Wang Z., Sun J., Huang Y., Hanif Q., Liao Y., Lei C. (2020). Identification of Genomic Characteristics and Selective Signals in a Du’an Goat Flock. Animals.

[60] Boggild A.K., Caumes E., Grobusch M.P., Schwartz E., Hynes N.A., Libman M., Connor B.A., Chakrabarti S., Parola P., Keystone J.S. (2019). Cutaneous and mucocutaneous leishmaniasis in travellers and migrants: A 20-year GeoSentinel Surveillance Network analysis. J. Travel Med..

[61] Demers E., Forrest D.M., Weichert G.E. (2013). Cutaneous leishmaniasis in a returning traveller. CMAJ.

[62] Riebenbauer K., Czerny S., Egg M., Urban N., Kinaciyan T., Hampel A., Fidelsberger L., Karlhofer F., Porkert S., Walochnik J. (2024). The changing epidemiology of human leishmaniasis in the non-endemic country of Austria between 2000 to 2021, including a congenital case. PLoS Neglected Trop. Dis..

[63] Meng Z., Fan P.W., Fan Z.X., Chen S., Jiang H., Shi Y., Yao L., Yao J.Y., Wang Y.P., Hao M.M. (2025). Environmental change increases the transmission risk of visceral leishmaniasis in central China around the Taihang mountains. Environ. Health.

[64] Rodrigues M.G.A., Sousa J.D.B., Dias Á.L.B., Monteiro W.M., Sampaio V.S. (2019). The role of deforestation on American cutaneous leishmaniasis incidence: Spatial-temporal distribution, environmental and socioeconomic factors associated in the Brazilian Amazon. Trop. Med. Int. Health.

[65] González C., Wang O., Strutz S.E., González-Salazar C., Sánchez-Cordero V., Sarkar S. (2010). Climate change and risk of leishmaniasis in north america: Predictions from ecological niche models of vector and reservoir species. PLoS Neglected Trop. Dis..

[66] Senanayake S.C., Liyanage P., Pathirage D.R.K., Siraj M.F.R., De Silva B., Karunaweera N.D. (2024). Impact of climate and land use on the temporal variability of sand fly density in Sri Lanka: A 2-year longitudinal study. PLoS Neglected Trop. Dis..

[67] Muñoz Morales D., Suarez Daza F., Franco Betancur O., Martinez Guevara D., Liscano Y. (2024). The Impact of Climatological Factors on the Incidence of Cutaneous Leishmaniasis (CL) in Colombian Municipalities from 2017 to 2019. Pathogens.

[68] Bounoua L., Kahime K., Houti L., Blakey T., Ebi K.L., Zhang P., Imhoff M.L., Thome K.J., Dudek C., Sahabi S.A. (2013). Linking climate to incidence of zoonotic cutaneous leishmaniasis (L. major) in pre-Saharan North Africa. Int. J. Environ. Res. Public. Health.

[69] Balaska S., Calzolari M., Grisendi A., Scremin M., Dottori M., Mavridis K., Bellini R., Vontas J. (2023). Monitoring of Insecticide Resistance Mutations and Pathogen Circulation in Sand Flies from Emilia-Romagna, a Leishmaniasis Endemic Region of Northern Italy. Viruses.

[70] Vaselek S. (2021). Systematic Review: Re-emergence of human leishmaniasis in the Balkans. Trop. Med. Int. Health.

[71] Brilhante A.F., Zampieri R.A., Souza E.A., Carneiro A.C.G., Barroso E.P., Ávila M.M., Melchior L.A.K., Souza J.L., Oliveira E.S., Pinto M.C.G. (2022). Preliminary observations of the urbanization and domiciliation of the American cutaneous leishmaniasis in Rio Branco, Acre, Western Amazon. Rev. Soc. Bras. Med. Trop..

[72] Vadmal G.M., Glidden C.K., Han B.A., Carvalho B.M., Castellanos A.A., Mordecai E.A. (2023). Data-driven predictions of potential Leishmania vectors in the Americas. PLoS Neglected Trop. Dis..

[73] Shahryari A., Charkazi A., Rajabi A. (2023). Environmental factors and building conditions for risk of cutaneous leishmaniasis in the northeast of Iran: A population-based case-control study. Trans. R. Soc. Trop. Med. Hyg..

[74] Ardic I.N., Ardic N. (2023). A minor emphasis on the outbreak of cutaneous leishmaniasis after devastating earthquakes in Turkey. Asian Pac. J. Trop. Med..

[75] Sharifi I., Poursmaelian S., Aflatoonian M.R., Ardakani R.F., Mirzaei M., Fekri A.R., Khamesipour A., Parizi M.H., Harandi M.F. (2011). Emergence of a new focus of anthroponotic cutaneous leishmaniasis due to Leishmania tropica in rural communities of Bam district after the earthquake, Iran. Trop. Med. Int. Health.

[76] Aflatoonian M., Sharifi I., Aflatoonian B., Salarkia E., Khosravi A., Tavakoli Oliaee R., Bamorovat M., Aghaei Afshar A., Babaei Z., Sharifi F. (2022). Fifty years of struggle to control cutaneous leishmaniasis in the highest endemic county in Iran: A longitudinal observation inferred with interrupted time series model. PLoS Neglected Trop. Dis..

[77] Ambraseys N.N. (2001). Reassessment of earthquakes, 1900–1999, in the Eastern Mediterranean and the Middle East. Geophys. J. Int..

[78] Alharazi T.H., Haouas N., Al-Mekhlafi H.M. (2021). Knowledge and attitude towards cutaneous leishmaniasis among rural endemic communities in Shara’b district, Taiz, southwestern Yemen. BMC Infect. Dis..

[79] Rocha R., Pereira A., Maia C. (2022). Non-Endemic Leishmaniases Reported Globally in Humans between 2000 and 2021-A Comprehensive Review. Pathogens.

[80] Sunyoto T., Verdonck K., El Safi S., Potet J., Picado A., Boelaert M. (2018). Uncharted territory of the epidemiological burden of cutaneous leishmaniasis in sub-Saharan Africa-A systematic review. PLoS Neglected Trop. Dis..

[81] Pasquier G., Demar M., Lami P., Zribi A., Marty P., Buffet P., Desbois-Nogard N., Gangneux J.P., Simon S., Blaizot R. (2022). Leishmaniasis epidemiology in endemic areas of metropolitan France and its overseas territories from 1998 to 2020. PLoS Neglected Trop. Dis..

[82] Okwor I., Uzonna J. (2016). Social and Economic Burden of Human Leishmaniasis. Am. J. Trop. Med. Hyg..

[83] Maia-Elkhoury A.N.S., Yadón Z.E., Díaz M.I.S., de Araújo Lucena F.d.F., Castellanos L.G., Sanchez-Vazquez M.J. (2016). Exploring Spatial and Temporal Distribution of Cutaneous Leishmaniasis in the Americas, 2001–2011. PLoS Neglected Trop. Dis..

[84] Maia-Elkhoury A.N.S., SY O.B.V., Puppim-Buzanovsky L., Rocha F., Sanchez-Vazquez M.J. (2017). SisLeish: A multi-country standardized information system to monitor the status of Leishmaniasis in the Americas. PLoS Neglected Trop. Dis..

[85] Mikhail Ejov D.D. Strategic framework for leishmaniasis control in the WHO European Region 2014–2020. https://iris.who.int/bitstream/handle/10665/329477/9789289050166-eng.pdf?sequence=1&isAllowed=y.

[86] Saadene Y., Salhi A. (2025). Spatio-temporal modeling of Cutaneous Leishmaniasis under climate change scenarios in the Maghreb region (2021–2100). Acta Trop..

[87] Fournet F., Jourdain F., Bonnet E., Degroote S., Ridde V. (2018). Effective surveillance systems for vector-borne diseases in urban settings and translation of the data into action: A scoping review. Infect. Dis. Poverty.

[88] Bamorovat M., Sharifi I., Khosravi A., Aflatoonian M.R., Agha Kuchak Afshari S., Salarkia E., Sharifi F., Aflatoonian B., Gharachorloo F., Khamesipour A. (2024). Global Dilemma and Needs Assessment Toward Achieving Sustainable Development Goals in Controlling Leishmaniasis. J. Epidemiol. Glob. Health.

